# PERSONAL CARE PRODUCTS: Triclosan Comes under Scrutiny

**DOI:** 10.1289/ehp.118-a242

**Published:** 2010-06

**Authors:** Catherine M. Cooney

**Affiliations:** **Catherine M. Cooney**, a science writer in Washington, DC, has written for *Environmental Science & Technology* and Greenwire

Triclosan, the antimicrobial agent marketed for its germ-fighting capability in personal care products, is coming under close scrutiny. In April 2010 the U.S. Food and Drug Administration (FDA) announced it is conducting a scientific and regulatory review of triclosan in FDA-regulated products, with publication of results expected in spring 2011.^1^ The agency also is collaborating with the U.S. Environmental Protection Agency (EPA) specifically to study the potential endocrine-disrupting effects of the compound.^2^

Triclosan is a broad-spectrum antimicrobial agent developed over 40 years ago and first introduced as a surgical scrub. Over the last 20 years its use has grown rapidly in personal care products including soap, hand sanitizer, cosmetics, and toothpaste, as well as household products such as odor-fighting socks and germ-resistant sponges, kitchenware, and bedding. A 2001 U.S. study—the latest such data—found triclosan in 76% of 395 commercial soaps examined.^3^ In 2008 the Environmental Working Group reported finding triclosan in more than 140 types of personal care and home products.^4^ Data from the 2003–2004 National Health and Nutrition Examination Survey showed triclosan in 75% of urine samples analyzed.^5^ Triclosan also has been found in rivers and streams^6^ and in sewage sludge applied to agriculture.^7^

In January 2010, congressman Edward J. Markey (D–MA) wrote to the FDA and the EPA, urging the agencies to regulate triclosan and the biocide triclocarban.^8^ However, future regulation of triclosan in the United States is far from certain. “The efficacy of triclosan-containing products in household and other nonhealthcare-related settings and the potential hazards associated with [these uses] are the subject of an ongoing scientific debate,” says Antonia Calafat, lead research chemist in the Organic Analytical Toxicology Branch of the National Center for Environmental Health, Centers for Disease Control and Prevention.

One area of debate involves the hypothesis that triclosan enhances the production of chloroform, which is classified by the EPA as a probable human carcinogen. A study published in 2007 illustrated that, under some circumstances, triclosan triggered the production of chloroform in amounts up to 40% higher than background levels in chlorine-treated tap water.^9^ But another study published the same year showed no formation of detectable chloroform levels over a range of expected tooth-brushing durations among subjects using toothpaste with triclosan and normal chlorinated tap water.^10^

Studies also have yielded conflicting findings regarding links between triclosan and adverse health effects in animals. One study, for example, associated exposure to low levels of triclosan with disrupted thyroid hormone–associated gene expression in tadpoles, which encouraged them to prematurely change into frogs,^11^ while another linked triclosan exposure with reduced sperm production in male rats.^12^ In contrast, research published in February 2010 showed no effect of triclosan on the normal course of thyroid-mediated metamorphosis in bullfrog tadpoles at environmentally relevant concentrations.^13^

There is still less certainty about the potential for harmful effects in humans. A multiethnic longitudinal study of 1,151 U.S. girls identified small inverse associations for triclosan and high-molecular-weight phthalates with pubic hair stage, although the authors noted that “some or all of our findings may be due to chance.”^14^ The FDA notes the compound currently is not known to be hazardous to humans, and, moreover, the agency does not currently recommend any changes to consumer use of triclosan-containing products.^1^

ScientistsinEuropearelesssanguine.In2009 the European Union’s Scientific Committee on Consumer Products, which provides the European Commission (EC) with scientific advice, wrote that the toxicologic data suggest “the continued use of triclosan as a preservative at the current concentration limit of maximum 0.3% in all cosmetic products is not safe for the consumer because of the magnitude of the aggregate exposure.”^15^ However, the committee noted that continued use in specific subcategories including toothpaste, soap, deodorant, face powder, and blemish concealer is considered safe. In March the EC called for an assessment of whether triclosan in cosmetic products can lead to the development of resistance by certain microorganisms.^16^

One manufacturer already asked European regulators to withdraw its application for the use of triclosan in plastic products that come into contact with food. In a 2009 letter to the EC, officials from Ciba Inc. wrote that the company “does not consider the use of the substance in plastics intended to come into contact with food appropriate any more.”^17^

As for whether triclosan actually improves products to which it’s added, the FDA asserts it has no clear evidence that triclosan in antibacterial soaps and body washes provides extra health benefits over washing with regular soap and water, although it acknowledged evidence that triclosan in toothpaste may help prevent gingivitis.^1^ Paul DeLeo, senior director of environmental safety at The Soap and Detergent Association, maintains that antibacterial soaps with triclosan have been shown to work better than soap and water when killing harmful bacteria, citing a study by researchers from The Dial Corporation.^18^

DeLeo notes that years’ worth of research demonstrates the environmental safety of triclosan as reflected in studies showing that 90–98% of the compound is typically removed by wastewater treatment plants. However, says Rolf Halden, an associate professor in the School of Sustainable Engineering and the Built Environment at Arizona State University, although triclosan may be effectively removed from wastewater, only about 50% is degraded during treatment.^19^ “We are in a conundrum,” he says, “where we have all of this old data that shows that triclosan is safe, and all of this new data that shows the potential harm.” The challenge for the FDA and the EPA will be to figure out where the new data and the old intersect.

## Figures and Tables

**Figure:**
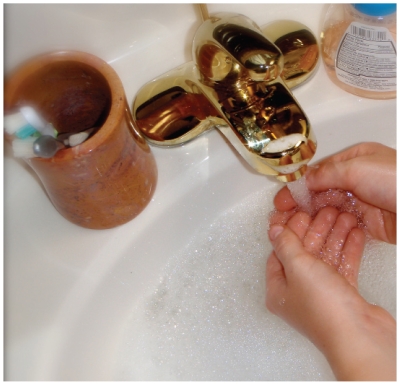
Federal agencies are reviewing triclosan’s safety but haven’t called for altered usage.

## References

[1] FDA (2010). Triclosan: What Consumers Should Know.

[2] EPA (2010). Triclosan Facts.

[3] Perencevich EN (2001). Am J Infect Control.

[4] EWG (2008). Pesticide in Soap, Toothpaste and Breast Milk—Is It Kid-Safe?.

[5] Calafat AM (2008). Environ Health Perspect.

[6] Halden RU, Paull DH (2005). Environ Sci Technol.

[7] Heidler J, Halden RU (2009). J Environ Monit.

[b8-ehp-118-a242] 8The letters are available at http://markey.house.gov/docs/fdatriclo.pdf and http://markey.house.gov/docs/epatriclo.pdf [accessed 13 May 2010].

[9] Fiss EM (2007). Environ Sci Technol.

[10] Hao Z (2007). Intl J Cosmetic Sci.

[11] Veldhoen N (2006). Aquat Toxicol.

[12] Kumar V (2009). Reprod Toxicol.

[13] Fort DJ (2010). Toxicol Sci.

[14] Wolff MS Environ Health Perspect.

[15] SCCS Request for a Scientific Opinion: Triclosan (CAS 3380-34-5) (EINECS 222-182-2) Supplement I (P32).

[16] (2010). Public Consultation on the SCCS Preliminary Opinion on the Antimicrobial Resistance Effect of Triclosan.

[17] Council Decision (EC) No 1613/2003, O.J. L 75 of 23 March 2010.

[18] Fischler GE (2007). J Food Protect.

[19] Heidler J, Halden RU (2007). Chemosphere.

